# Universal test and treat in Cameroon: a comparative retrospective analysis of mortality and loss to follow-up before and after a strategic change in approach to HIV care

**DOI:** 10.11604/pamj.2023.45.191.40448

**Published:** 2023-08-31

**Authors:** Cavin Epie Bekolo, Sylvester Atanga Ndeso, Linda Lucienne Moifo, Nkwele Mangala, Tatiana Danielle Yimdjo, Jerome Ateudjieu, Charles Kouanfack, Alain Djam, Earnest Njih Tabah, Solange Whegang, Clarisse Mapa-Tassou, Nicolas Tendongfor, Dickson Shey Nsagha, Siméon-Pierre Choukem

**Affiliations:** 1 Department of Public Health, Faculty of Medicine and Pharmaceutical Sciences, University of Dschang, Dschang, Cameroon,; 2 Department of Public Health and Hygiene, Faculty of Health Sciences, University of Buea, Buea, Cameroon,; 3 Department of Gynaecology and Obstetrics, University of Douala, Douala, Cameroon,; 4 Military Medical Centre, RASS, Nkongsamba, Cameroon,; 5 Department of Internal Medicine and Specialities, Faculty of Medicine and Pharmaceutical Sciences, University of Dschang, Dschang, Cameroon

**Keywords:** HIV, universal test and treat, loss to follow-up, mortality, Cameroon

## Abstract

**Introduction:**

an increasing number of persons living with HIV (PLHIV) are accessing antiretroviral therapy (ART) since the adoption of the universal test and treat (UTT) policy by Cameroon in 2016. We sought to evaluate the effectiveness of the UTT approach to keep this growing number of PLHIV on a lifelong treatment.

**Methods:**

a retrospective cohort analysis was conducted at the Nkongsamba Regional Hospital between 2002 and 2020, using routine data to compare the cumulative incidence of loss to follow-up (LTFU) and mortality between PLHIV initiated on ART under UTT guidelines and those initiated under the standard deferred approach. Chi-squared test was used to compare the risk of attrition between the guideline periods while multiple logistic regression modelling was used to adjust for confounders.

**Results:**

of 1627 PLHIV included for analysis, 756 (46.47%) were enrolled during the era of UTT with 545 (33.54%) initiated on ART on the same day of HIV diagnosis. The transition to the UTT era was associated with an overall reduction in the risk of LTFU by 73% (aOR = 0.27, 95%CI: 0.17 - 0.45). There was modest evidence that the odds of mortality had increased under the UTT policy by about 3-fold (aOR = 2.86, 95%CI: 0.91-8.94). Same-day initiation had no overall effect on LTFU or mortality. LTFU was lower among the same-day initiators in the first 24 months but increased thereafter above the rate among late initiators.

**Conclusion:**

overall ART programme implementation under the UTT has led to a significant decline in LTFU though mortality appeared to have increased. Ongoing efforts to keep patients on long-term treatment should be sustained while other innovative schemes are sought.

## Introduction

Cameroon with approximately 500 000 people living with the Human Immunodeficiency Virus (HIV) adopted and started implementing in 2016, the ‘universal test and treat’ (UTT) policy to enhance progress towards the ambitious 95-95-95 global targets to end the epidemic by 2030 [1,2]. As per the World Health Organization (WHO) 2015 UTT guidelines, the CD4 cell count threshold for ART eligibility was removed making all HIV-positive patients eligible for antiretroviral therapy (ART) at diagnosis [3]. Consequently, the UTT policy led to a significant increase in the number of persons accessing ART from 168431 (27.1% ART coverage) in 2015 to 350818 (70.6% ART coverage) in 2020 [4]. While this number is expected to increase rapidly, there are concerns over the country´s health system's capacity to absorb and maintain the growing numbers of clients on lifelong ART. ART has transformed HIV/AIDS into a chronic illness and a lifelong follow-up on ART is mandatory for optimal outcomes. Loss of patients to follow-up (LTFU) is, however, an important problem in resource-limited settings and is associated with high mortality [5-8]. The increasing patient load under the UTT policy is likely to further stretch the already weakened health system and consequently lead to further LTFU in HIV care. A systematic review of clinical trials that informed the UTT guidelines however demonstrated that retention rates increased by 12% and mortality rates were reduced by 53% after 12 months of follow-up [9]. As countries scale up the implementation of UTT policy, evidence of the real-world impact of UTT on loss to follow-up (LTFU) is emerging but contradictory. Seven studies from China, Zambia, Malawi, Thailand, Ethiopia, and Indonesia reported favourable retention and survival rates of implementing UTT [10-15]. Meanwhile, 12 studies from relatively high-burdened countries including Eswatini, South Africa, Nigeria, Zimbabwe, Uganda, and Haiti indicated that UTT policy was associated with higher LTFU and death rates [16-27]. A systematic review of observational studies indicated no evidence that UTT was associated with a greater likelihood of being lost to follow-up (pooled RR: 1.85, 95% CI 0.96-3.55) [3]. These differences are because these studies highly differ in their social, demographic, economic, political, and health systems settings. They also differ in methodological aspects such as study design, eligibility criteria, and endpoints or outcome measures. These differences in context and approaches have led to inconsistent outcomes and thus created an implementation gap, and as such, each setting needs to determine its outcomes and institute improvements in its HIV programme. These outcomes are unknown to the ART programme in Cameroon with a pre-UTT retention rate of 75% in 2015 [28]. Monitoring the quality of HIV care through attrition from the expanding ART cohorts and examining whether this has improved evidence-based clinical management, are important goals of policy implementation and evaluation in Cameroon. We aim to evaluate the effect of this novel approach on the quality of care and health outcomes of persons living with HIV (PLHIV) by comparing the cohort who started ART under the UTT approach with a cohort who initiated ART under the previous deferred treatment approach. Specifically, we sought to look at the changes in LTFU and mortality rates after the introduction of the UTT policy in Cameroon; and to compare LTFU and mortality rates between same-day and delayed ART initiators. We hope this evaluation would be an essential tool for determining how well the test and treat programme in real-life practice is performing to meet the needs of service users and assessing whether improvements are required.

## Methods

**Setting:** the study was conducted at the Regional Hospital of Nkongsamba in the Mungo Division of the Littoral Region of Cameroon. It is a second-level reference public health facility with a catchment area of over 321,295 inhabitants [29]. The clinic was established in 2005 and offers voluntary HIV counselling and testing (VCT), ART and limited community outreach services to over 2000 patients on ART. HIV services are provided by multidisciplinary teams composed of physicians, health officers, nurses, pharmacy attendants, laboratory technicians, psychosocial/adherence supporters, and data personnel. Before UTT, amongst the 1858 enrolled since 2005, 192(10.3%) were confirmed dead, 680(36.6%) had been LTFU, and 165(8.9%) were confirmed to have been transferred out by June 2012 [5]. As of 2016, per the national ART guideline, PLHIV were immediately linked to an ART clinic for a confirmatory test, counselling, adherence preparation and rapid ART initiation - including same-day ART for persons who are ready to start ART at the first clinical visit in the absence of CD4 testing that would otherwise delay ART initiation.

**Study design:** we conducted in 2022, a facility-based retrospective cohort analysis of routinely collected clinical service delivery data abstracted from facility-based medical records. All PLHIV aged 15 and above enrolled on ART care between 2002 and 2020 were eligible for inclusion. From the chart review, PLHIV enrolled up to the year 2016 were included in the pre-UTT group while those enrolled after the year 2006 were included in the UTT group. Based on days from HIV+ diagnosis to ART initiation, PLHIV were further stratified into the same-day initiation (SDI) group, rapid initiation group (1-7 days) or the deferred initiation group (8+ days). The groups were then followed up retrospectively until they either died or were lost to follow-up.

**Study size calculation:** the sample size was determined using G*Power 3.1 (Universität Kiel, Germany) for cohort studies based on the difference in the proportion of retention in care. We used the proportion of retention at 12 months among PLHIV initiated under standard care based on a nationally representative study in 2015 and then estimated a 10% increase in retention at 12 months in the test and treat group [28]. Accordingly, P1 of 75% (proportion of retention at 12 months of ART among persons in the standard group), P2 of 85% (proportion of retention at 12 months of ART among individuals in the test and treat group), a 1: 1 proportion, of 0.05, and 95% power was used to calculate the sample size of 826 (413 per group) participants. We increased the required sample size by 20% to account for anticipated limitations regarding missing medical record data. Based on this, we estimated that we would need to enrol a minimum of 976 participants.

**Data collection and analysis:** we used data routinely collected from individual patient medical records designed by the National AIDS Control Committee (NACC) for the standardised collection and reporting of data. We extracted the following study variables using a data extraction form: socio-demographic characteristics: date of birth, gender, place of residence, occupation, alcohol and tobacco consumption, and matrimonial status; clinical features including date of HIV diagnosis, and WHO clinical stage at presentation, baseline CD4 count; treatment-related variables including date of ART initiation, ART regimen, adherence, cotrimoxazole preventive treatment (CPT) and isoniazid prevention therapy (IPT).

**Data analysis:** data collected were exported to Stata® 15.1 (StataCorp LLC, Texas 77845, USA) for statistical analysis. The data set was explored for logical inconsistencies, illegal codes, omissions and improbabilities by tabulating, summarising, describing, and plotting variables. Missing observations were excluded where they constituted a small random proportion. Summary statistics were presented as proportions for categorical variables and as means (standard deviations) for normally distributed continuous variables or medians (IQR-Interquartile Range) for skewed distributed continuous variables. Our main outcomes of interest were all-cause deaths and losses to follow-up (LTFU). Death was defined as all-cause death according to the patient´s medical record or ART register. Individuals who did not refill their ART for a period of 90 days or more after their last refill appointment date and were not yet classified as having died or transferred out were labelled as LTFU. The main explanatory variable of interest was the exposure to the UTT strategy. A logistic regression model was set up to assess the association between UTT strategy and attrition from care and to screen for factors associated with attrition. Crude odd ratios (OR) and their 95% confidence intervals (CI) were obtained. The p-values for hypotheses testing were calculated from Wald or likelihood ratio tests (LRT). Variables found to be associated at a 5% confidence level, with attrition were included in a multivariable logistic regression model. Backwards elimination based on a p-value lower than 0.05 was used to retain variables independently associated with attrition. The corresponding adjusted odd ratios (aOR), their 95% confidence intervals and p-values in the final model were reported. We estimated the predictive margins of LTFU for each guideline period and their interaction with days to ART initiation.

**Ethics approval and consent to participate:** ethical approval was obtained from The Littoral Regional Ethics Committee for Research in Humans in Cameroon. Permission to use data was duly obtained from the hospital management board. Consent from individual patients was not sought because we used routine data. However, all patient information was anonymised and de-identified before analysis. Access to the database was protected by a password.

## Results

**Baseline characteristics of participants:** a total of 1651 persons diagnosed HIV positive between 2002 and 2020 were identified, of whom 1627 with identifiable data were included for analysis. Of those included, 756 (46.47%) were enrolled during the era of UTT with 545 (33.54%) initiated on ART on the same day of HIV diagnosis (Table 1). Participants enrolled under the UTT era were more likely to start ART on the same day of their HIV diagnosis (67.24%), to be married (35.16%), be living close to the HIV clinic (50.07%) or currently be in employment (68.98%). Baseline CD4 count testing was practically phased out during the UTT era (0.93%) and where available, it was targeted to those who were likely to have a titre below 350 cells/mm^3^ but late clinical presentation was uncommon under UTT (17.56%).

**Table 1:** baseline characteristics of the study population by the period of ART initiation

Variables	Before UTT	Under UTT
	n (%)	n (%)
Time to ART initiation		
Same-day initiation (SDI)	38(4.36)	507(67.24)
Rapid initiation (RI)	135(15.50)	142(18.83)
Deferred initiation (DI)	698(80.17)	105(13.93)
**Total**	871(100)	754(100)
Age group(years)		
<=40	419 (48.44)	350 (46.42)
>40	446(51.56)	404(53.58)
Total	865 (100)	754 (100)
**Sex**		
Female	576(66.63)	527(70.36)
Male	287(33.37)	222(29.64)
Total	860(100)	749(100)
Marital status		
Single	388 (46.92)	344 (47.25)
Married	245(29.63)	256(35.16)
Divorced	51(6.17)	35 (4.81)
Widowed	143 (17.29)	93(12.77)
**Total**	827(100)	728(100
Residence		
Local	306(35.75)	374 (50.07)
Regional	463 (54.09)	297 (39.76)
Inter-regional	87 (10.16)	76(10.17)
Total	856(100)	747(100)
**Occupation**		
None	318 (36.98)	215 (29.78)
Currently employed	536(62.33)	498(68.98)
Retired	6(0.70)	9(1.25)
**Total**	860 (100)	722 (100)
Baseline CD4 testing		
No	46 (5.31)	749 (99.07)
Yes	821 (94.69)	7 (0.93)
**Total**	867 (100)	756 (100)
CD4 count per mm^3^		
<350	442(53.90)	5(83.33)
>=350	378(46.10)	1(16.67)
Total	820 (100)	6 (100)
**WHO Clinical Stage**		
Early (stages I & II)	335 (48.83)	582 (82.44)
Advanced (stages III & IV)	351 (51.17)	124(17.56)
**Total**	686(100)	706(100)
Smoking		
No	768(88.17)	676(89.42)
Yes	103 (11.83)	80 (10.58)
**Total**	871 (100)	756 (100)
**Alcohol intake**		
**No**	415 (47.65)	311 (41.14)
**Yes**	456(52.35)	445(58.86)
**Total**	871(100)	756(100)

**Figure 1:**
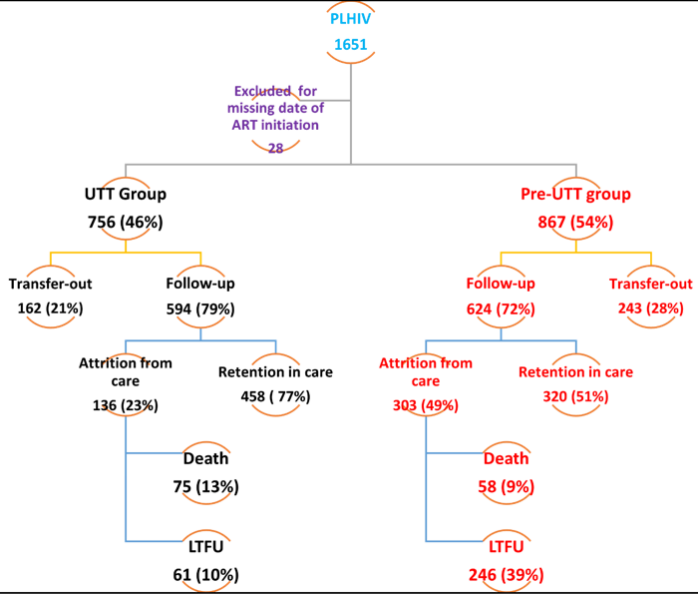
the cascade of HIV care across treatment guidelines

**Figure 2:**
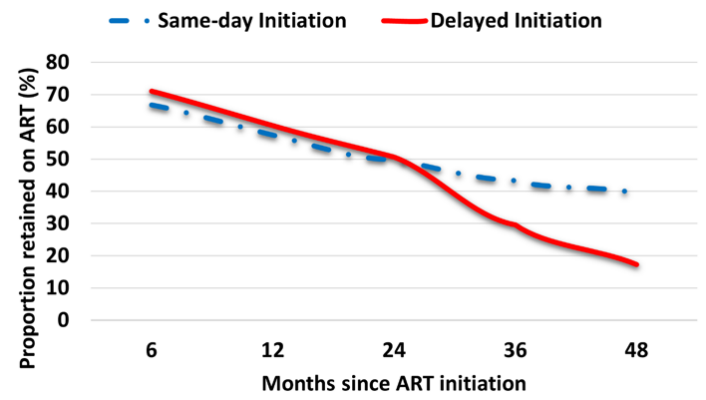
predicted probabilities of loss to follow-up before and after implementation of UTT policy

**Loss to follow-up before and after the introduction of ‘universal test and treat’:** a total of 307 PLHIV were lost to follow-up computing to a cumulative incidence of 25.12% (95%CI: 22.72-27.64). The risk of LTFU dropped significantly from the pre-UTT era of 39.42% to 10.27% (p<0.001). Similarly, retention in care after accounting for transferred outs, increased by 25.74% from the pre-UTT period of 51.36% to 77.10% under the UTT policy (Figure 1). There was a linear increase in LTFU rate as we moved from same-day initiation (7.34%) across rapid initiation (19.86%) to deferred initiation (26.28%) depicting a trend or dose-response relationship between LTFU and time to ART initiation. This relationship was true during the first 24 months of follow-up but afterwards, SDI was associated with increased LTFU (Figure 2). After adjusting for confounding, the transition to the UTT guidelines was associated with a reduction in the risk of LTFU by 73% (aOR = 0.27, 95%CI: 0.17- 0.45). PLHIV enrolled under the UTT policy had consistently lower probabilities of being LTFU regardless of time spent between HIV diagnosis and ART initiation (Figure 2). There was no evidence to support the claim that time to ART initiation was associated with LTFU. Other factors independently associated with a lower risk of LTFU were early clinical presentation and single marital status (Table 2).

**Table 2:** factors associated with loss to follow-up in a logistic regression model

Factor	Risk of LTFU n (%)	Crude Odd Ratio (95%CI)	Adjusted Odd Ratio (95%CI)
**Cohort**			
Before UTT	246 (28.37)	1	1
Under UTT	61(8.01)	0.18(0.13-0.24)	0.27(0.17-0.45)
**ART initiation strategy**			
SDI	40(9.71)	0.19 (0.13-0.28)	0.74 (0.42-1.28)
Rapid initiation	55(25.58)	0.62 (0.44-0.88)	1.08 (0.69-1.69)
Deferred	210(35.65)	1	1
**WHO Clinical Stage**			
Early (stages I & II)	120(16.76)	1	1
Advanced (stages III & IV)	141(40.29)	3.35(2.51-4.48	2.10(1.51-ï¿½2.92)
**Marital status**			
Single	171(31.03)	1	1
Married	53(13.98)	0.36(0.26-0.51)	0.34(0.23-0.51)
Divorced	25(34.25)	1.16(0.69-1.94)	0.79(0.42 -1.50)
Widowed	43(24.86)	0.74(0.50-1.08)	0.65 (0.41-1.03)

**Mortality before and after the introduction of ‘universal test and treat’:** a total of 135 deaths were recorded computing a cumulative incidence of 10.94% (95%CI: 9.25-12.81). The risk of mortality increased from 9.29% in the pre-UTT era to 12.63% under the UTT policy (p = 0.062). There was no association between the risk of death and time to ART initiation (Table 3). After controlling for covariates in a multivariable logistic regression model, there was still just a little evidence to support the finding that the odds of mortality had increased under the UTT policy by about 3-folds (aOR= 2.86, 95%CI: 0.91- 8.94). The main determinants of mortality were presentation with advanced HIV disease that was associated with approximately 4-fold the likelihood of death (aOR = 3.88, 95%CI: 1.59-9.46) and a suppressed viral load that was associated with a reduction of the likelihood of death by 84% (aOR = 0.16, 95%CI: 0.06- 0.38).

**Table 3:** factors associated with mortality in a logistic regression model

Factor	Risk of Death n (%)	Crude Odd Ratio (95%CI)	Adjusted Odd Ratio (95%CI)
**Cohort**			
Before UTT	58(6.69)	1	1
Under UTT	75(9.92)	1.53(1.07-2.20)	2.86(0.91-8.94)
**ART initiation strategy**			
SDI	42(10.19)	1	1
Rapid initiation	28 (13.02)	1.32 (0.79-2.19)	0.71 (0.18 ï¿½ 2.77)
Deferred	63(10.70)	1.05(0.70-1.59)	1.02(0.30-3.49)
**WHO Clinical Stage**			
Early(stages I & II)	50(6.98)	1	1
Advanced(stages III & IV)	61(17.43)	2.81 (1.89 -4.19)	3.88 (1.59-9.46)
**Viral load**			
High	19 (12.93)	1	1
Suppressed	14(2.54)	0.18(0.09-0.36)	0.16(0.06-0.38)

## Discussion

This observational study is one of the first studies in Cameroon and Central Africa that attempts to document the changes in loss to follow-up and mortality before and during the implementation of the “Universal Test and Treat” policy. The study has indicated that implementing UTT was associated with a 73% reduction in the likelihood of being LTFU in ART care. It also alleged that the odds of mortality had almost tripled under the UTT policy. No evidence was found of any effect of same-day initiation on attrition from ART care.

The observed decline in LTFU under the UTT policy is consistent with findings from clinical trials [9] that informed the UTT guidelines and from real-world studies in China, Zambia, Malawi, Thailand, Ethiopia, and Indonesia [10-15]. The findings are however contradictory to results reported from the implementation of UTT guidelines in Eswatini, South Africa, Nigeria, Zimbabwe, and Uganda [16-27]. A systematic review of observational studies conducted by WHO indicated no evidence that UTT was associated with a greater likelihood of being lost to follow-up (pooled RR: 1.85; 95% CI 0.96-3.55) [3]. Another review Conducted in sub-Saharan countries found no difference in ART attrition at 12 months before and after UTT in a meta-analysis of eight studies (pooled RR = 1.08; 95% CI: 0.97-1.21) [30]. There was very high heterogeneity among the studies, in terms of study populations and settings (social, demographic, economic, political, and health systems), the definition of UTT as SDI and/or rapid initiation or as an initiation in the period following the introduction of UTT policy, time frames, study design, endpoints and data analysis approaches. In Cameroon, we found two studies that reported a negative effect of UTT on retention. An analysis of the national ART database conducted between 2016 and 2017 indicated that retention in care dropped slightly from 77,2% to 75,5% [31]. This analysis had the merit of being nationally representative but was done during the transition period and was likely to produce suboptimal outcomes. Another study reported a similar decrease in retention from 85.79% to 78.83% after 24 months in three referral hospitals in Cameroon [32,33].

This study also selected their cohort during the transition period in 2016 and did not account for confounding in a multiple regression analysis. Our study was conducted after five years of UTT implementation and performed statistical analyses to account for the independent effect of UTT and related strategies. The decline in LTFU (increased in retention) observed in this study was not attributed to the test and treat strategy based on same-day and rapid initiation per se but probably to secular factors or interventions that were scaled up during the UTT era to strengthen the health system and thus reduce LTFU. These interventions were designed to address the factors we identified at the study site in 2012 to be related to the high LTFU rate of 36.6% before the UTT policy was adopted [5]. Two of these factors including a late presentation with advanced HIV disease and a married marital status have been identified to be associated with LTFU in the UTT era. These interventions based on differentiated care included decentralisation of ART care to take services closer to home and reduce caseload at the study site; task shifting to reduce workload per healthcare worker; enrolment of psychosocial and community-based organisations to improve patient engagement in care; use of more tolerable and potent drugs, multi-month drug dispensation and less frequent clinic visits to reduce workload and transport costs. However, we were not able to adjust for these time-dependent and system-related confounders in our analyses. Therefore, our findings suggest that keeping a PLHIV on a lifelong treatment does not depend on when he/she starts treatment after knowing his/her HIV status but on how he/she is cared for once treatment is initiated. Although retention in care has significantly improved under UTT, it remains suboptimal so much so that ongoing efforts to keep patients on long-term treatment should be sustained while other innovative schemes are sought.

Despite improved retention in care under UTT, cumulative mortality had instead increased though the evidence provided by our data to support this increase was weak. We demonstrated before UTT that mortality in this cohort was driven by LTFU because two-thirds of LTFU who were traced had died [5]. We equally demonstrated that as UTT uptake was scaling up, ART uptake and viral load suppression (VLS) rates had increased exponentially [34]. So, we expected mortality rates to decrease because of the decline in the LTFU rate and the increase in ART uptake and VLS. Yet, we observed that there was a remarkable decline in CD4 count testing at the baseline necessary to detect PLHIV who should benefit from a preventive care package against opportunistic infections. Consequently, there was a rise in the incidence of opportunistic infections under UTT [34]. We strongly believe that the purported rise in mortality could be attributed to this burden of opportunistic infections. Our study was not powered enough to detect the difference in mortality risks between pre-UTT and UTT groups because of the rare event (death) in the numerators and the differential exclusion of transferred outs in the denominators (28% vs. 21%, p = 0.002). There was a significant difference in mortality risks between the groups when persons transferred out were included in the denominators. However, we don´t know whether transferred-outs had a differential survival profile before and after the introduction of UTT. We recommend a large multicentre study to help overcome the limitations inherent to a small study to verify if similar mortality trends were to be observed elsewhere in the country.

## Conclusion

The universal test and treat policy implemented as same-day or rapid ART initiation had no effect on LTFU and mortality in this cohort of PLHIV. However, overall, ART programme implementation under the UTT policy has led to a significant decline in LTFU though mortality appeared to have increased. PLHIV should not be coerced to start ART immediately after HIV diagnosis if they are not ready but need patient-centred care delivered under UTT to be kept on lifelong ART.

### What is known about this topic

- *Clinical trials had demonstrated that retention rates increased, and mortality rates were reduced under the UTT policy;*
- *Implementation research had indicated conflicting results on the effect of UTT on loss to follow-up and mortality*.

### What this study adds

- *Loss to follow-up in ART care has improved significantly under the UTT policy;*
- *Mortality rate has increased non-significantly under the UTT policy;*
- *Same-day or rapid ART after HIV diagnosis has no effect on LTFU or mortality*.
